# Multiple Genes in a Single Host: Cost-Effective Production of Bacterial Laccase (cotA), Pectate Lyase (pel), and Endoxylanase (xyl) by Simultaneous Expression and Cloning in Single Vector in *E*. *coli*

**DOI:** 10.1371/journal.pone.0144379

**Published:** 2015-12-07

**Authors:** Sandeep Kumar, Kavish Kumar Jain, Kailash N. Bhardwaj, Subhojit Chakraborty, Ramesh Chander Kuhad

**Affiliations:** 1 Lignocellulose Biotechnology Laboratory, Department of Microbiology, University of Delhi South campus, Benito Juarez Road, New Delhi, 110021, India; 2 Uttarakhand State Council of Science and Technology [UCOST], Vigyan Dham, Post Office- Jhajra, Dehradun, Uttarakhand, 248007, India; 3 Central University of Haryana, Jant-Pali Village, Mahendergarh District, Pali, Haryana, 123029, India; Universidade Nova de Lisboa, PORTUGAL

## Abstract

This study attempted to reduce the enzyme production cost for exploiting lignocellulosic materials by expression of multiple genes in a single host. Genes for bacterial laccase (CotA), pectate lyase (Pel) and endoxylanase (Xyl), which hold significance in lignocellulose degradation, were cloned in pETDuet-1 vector containing two independent cloning sites (MCS). *CotA* and *xyl* genes were cloned in MCS1 and MCS 2, respectively. *Pel* gene was cloned by inserting complete cassette (T_7_ promoter, ribosome binding site, *pel* gene, His tag and complete gene ORF) preceded by *cotA* open reading frame in the MCS1. IPTG induction of CPXpDuet-1 construct in *E*. *coli* BL21(DE3) resulted in expression of all three heterologous proteins of ~65 kDa (CotA), ~45 kDa (Pel) and ~25 kDa (Xyl), confirmed by SDS-PAGE and western blotting. Significant portions of the enzymes were also found in culture supernatant (~16, ~720 and ~370 IU/ml activities of CotA, Pel and Xyl, respectively). Culture media optimization resulted in 2, 3 and 7 fold increased secretion of recombinant CotA, Pel and Xyl, respectively. Bioreactor level optimization of the recombinant cocktail expression resulted in production of 19 g/L dry cell biomass at OD_600nm_ 74 from 1 L induced culture after 15 h of cultivation, from which 9, 627 and 1090 IU/ml secretory enzyme activities of CotA, Xyl and Pel were obtained, respectively. The cocktail was also found to increase the saccharification of orange peel in comparison to the xylanase alone. Thus, simultaneous expression as well as extra cellular secretion of these enzymes as cocktail can reduce the enzyme production cost which increases their applicability specially for exploiting lignocellulosic materials for their conversion to value added products like alcohol and animal feed.

## Introduction

Rising cost of fossil fuels, their dwindling resources and increasing environmental concerns have motivated researchers to look for alternate liquid transportation fuels especially biofuels from plant biomass [1]. Degradation of the plant biomass using chemicals for this purpose is considered as uneconomic and unfriendly to the environment [2]. Use of biocatalyst such as cellulases, hemicellulases, pectinases and ligninases on the other hand is preferred due to cost effective production and less impact on the environment [1]. Hydrolysis of the lignocellulosic biomass for the production of fermentable sugars is catalyzed mainly by cellulases; however, due to complex structure of the biomass, accessibility of cellulose to these enzymes is hampered [2]. Hemicellulases, pectinases and laccases are key enzymes which facilitate the hydrolysis process by loosening lignocellulose structure [2]. In other words, depolymerization of plant biomass is achieved by synergistic action of cellulases, hemicellulases, pectinases and ligninases [3]. To improve the production of lignocellulolytic enzymes, various strategies have been adopted, including heterologous expression, use of strong promoter and manipulation of the metabolism of the organisms [4]. Till date, most of these enzymes have been produced individually by respective microorganisms employed and there are hardly any reports on the efficient production of all these enzymes by a single microorganism, which will also be cost effective for degradation of lignocellulosics. Besides, low level of production of these enzymes from wild type microorganisms also limits their utility for commercial exploitation.

Recently, combined expression of two or more enzymes using single or multiple plasmids in single host is generating extensive interest, however the major drawback in multiple plasmids based strategy is their maintenance by the host cells [5]. In single plasmid based approach, co-expression of multiple genes can be derived by cloning more than one gene with its expression assembly in a single plasmid [5]. Although attempts have been made to simultaneously express two lignocellulolytic enzymes [6,7], however, the possibility of simultaneous expression of more than two lignocellulolytic genes in a single host remain to be explored. Liu and Yu developed the process for fungal endoxylanase and β-glucosidase production as cocktail in *E*. *coli* by developing bi-cistronic vector based upon pET30 plasmid, which resulted in production of enzyme cocktail with enhanced activities [6]. Similarly, Fonseca-Maldonado et al. reported the production of bacterial xylanase and laccase with 44% enhanced activity as cocktail from *Pichia pastoris* expression system following homologous recombination based approach [7]. Co-expression of multiple heterologous proteins in same cell provides alternative way of avoiding *in vitro* reconstitution, reduction in possibilities of improper folding, ease of simultaneous purification and reduction in production cost [8].

Therefore, we have made an attempt to minimize the enzyme production cost following simultaneous expression of three bacterial enzymes, viz. spore coat A protein (CotA) and endoxylanase (Xyl) from *Bacillus pumilus* MK001 and pectate lyase (Pel) from *B*. *subtilis* RCK in *E*. *coli* BL21(DE3) by modifying pETDuet-1 vector. In addition to intracellular fraction, sub-cellular localization of these enzymes has also been investigated. Further, an attempt has also been made to scale up the recombinant enzyme cocktail production in 3.0 L bioreactor. In addition, lignocellulosic substrate hydrolyzing efficiency of the recombinant enzyme cocktail has also been tested on dried orange peel.

## Materials and Methods

### Bacterial strains, plasmids, restriction enzymes, antibiotics and other chemicals

*B*. *pumilus* MK001, *B*. *subtilis* RCK and different *E*. *coli* strains, BL21(DE3), BL21(DE3) containing pTUM4 plasmid (bears genes for proteins assisting in refolding) and *E*. *coli* BL21(DE3) arctic expression host, were obtained from Lignocellulose Biotechnology Laboratory, Department of Microbiology, University of Delhi South Campus, New Delhi. *B*. *subtilis* RCK and *B*. *pumilus* MK001 were maintained on Horikoshi agar media containing 0.5% glucose, 0.5% peptone, 0.5% yeast extract, 0.15% KH_2_PO_4_, 0.01% MgSO_4_.7H_2_O and 2.0% agar, pH 9.0. Fusion DNA polymerase, restriction endonuclease and T_4_ DNA ligase were procured from New England Biolabs Inc. (UK). Ampicillin and kanamycin antibiotics were obtained from HiMedia, India. Trypsin and Tris base were purchased from Sigma-Aldrich, USA, while remaining chemicals and media components of analytical grade were purchased locally.

### Synthesis of CPXpETDuet-1 plasmid

**Fig 1:**
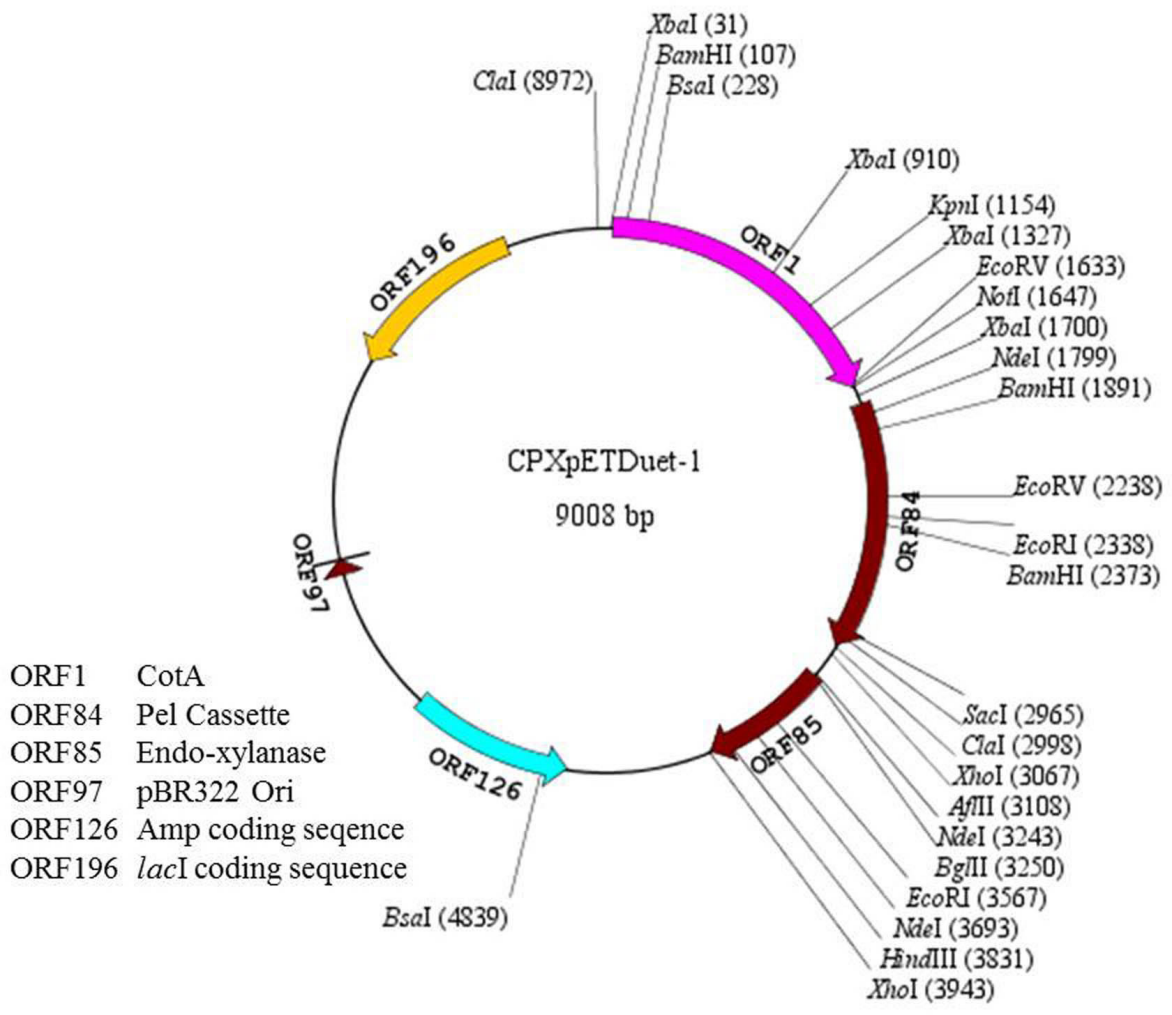
Vector map of CPXpETDuet-1 (Plotted using SimVector4.0 software).

Genomic DNA of *B*. *pumilus* MK001 and *B*. *subtilis* RCK was isolated following Murmur’s method [9]. Primers for CotA, Pel and Xyl encoding genes were designed using the DNA sequences available in the database (http://www.ncbi.nlm.nih.gov/gene) (Table 1). All three genes were amplified from the genomic DNA using gene specific primers, digested through respective restriction endonucleases and cloned individually in pET28a vector (Table 1). After analyzing the genes for the presence of complete ORF, they were amplified and cloned in pETDuet-1 using primers mentioned in Table 1. *CotA* gene was cloned at *Bam*HI-*Hin*dIII restriction sites in MCS1 while *xyl* gene was ligated at *Bgl*II-*Xho*I restriction site in MCS2 of the CotpETDuet-1. Contrary to *cotA* and *xyl*, *pel* gene was amplified from pET28a construct including T_7_ promoter, transcription start site and ribosome binding site (rbs) without nucleotide sequence for T_7_ terminator. The *pel* cassette was cloned at *Not*I-*Afl*II restriction sites in MCS1 preceded by *cotA* gene at 5’ terminus (S1 Fig). The constructed plasmid possessing all three genes was named as CPXpETDuet-1 (Fig 1).

**Table 1:** Primer sequences used for amplification of CotA, Pel and Xyl genes.

Primer	Sequence
CotA_*Bam*HI_pET28F	5’GCG GGATCC TATGAACCTAGAAAAATTTGTTGAC3’
CotA_*Xho*I_pET28R	5’CATA CTCGAG TTACTGGATGATATCCATCGGC3’
Xyl_ *Bam*HI_pET28F	5’CTA GGATCC ATGAATTTGAAAAGATTGAGGCTGTTGT3’
Xyl_ *Xho*I_pET28R	5’CATA CTCGAG ATGAATTTGAAAAGATTGAGGCTG3’
Pel_*Nde*I_pET28F	5’CTA CATATG AAAAAAGTGATGTTAGCTACGGC 3’
Pel_*Xho*I_pET28R	5’ CTA CTCGAG TTAATTTAATTTACCCGCACCCGC 3’
CotA_*Bam*HI_pDuetF	5’GCG GGATCC TATGAACCTAGAAAAATTTGTTGAC3’
CotA_*Hin*dIII_pDuetR	5’CATA AAGCTT TTACTGGATGATATCCATCGGC3’
Xyl_*Bgl*II_pDuetF	5’CTA GGTACC ATGAATTTGAAAAGATTGAGGCTGTTGT3’
Xyl_*Xho*I_pDuetR	5’CATA CTCGAG ATGAATTTGAAAAGATTGAGGCTG3’
PelCass_*Not*I_pDuetF	5’CTA CGCCGGCG TAATACGACTCACTATAGGGGAATTGT3’
PelCass_*Afl*II_pDuetR	5’ CTA CTTAAG TTAGCAGCCGGATCTCAGTGGTGGTGG3’

### Optimization of expression media and host

CPXpETDuet-1 plasmid was transformed into *E*. *coli* BL21(DE3) ultra-competent cells. Initially, expression was carried out in LB medium for the confirmation of recombinant enzyme cocktail production. Primary culture was prepared by single transformant colony inoculation in 15 ml LB ampicillin (100 μg/ml) broth and subsequent incubation at 37°C, 200 rpm. Fresh sterile 15 ml LB broth was inoculated with 2% primary culture and incubated at 37°C, 200 rpm till OD_600nm_ reached 0.7. At this OD, the culture was induced with 0.6 mM IPTG. The culture medium was also supplemented with CuCl_2_ (0.25 mM) as cofactor for CotA. An un-induced culture containing recombinant plasmid, CPXpETDuet-1, served as control. The induced culture was incubated at 25°C, 200 rpm for initial 8 h and thereafter kept static for next 16 h for proper incorporation of Cu^2+^ ions in CotA structure. Samples were collected at regular time intervals to determine activities of all three enzymes in culture supernatant (CS), periplasmic (PF) and cytoplasmic fractions (CF). Expression was confirmed by western blot using anti His antibodies.

Further to select optimum growth medium for maximum production of enzymes, expression of CPXpETDeut-1was carried out separately in different cultivation media viz. (i) LB broth, (ii) LB supplemented with 1% (w/v) wheat bran and (iii) terrific broth (TB) using same method as described in last paragraph. Values of the physical parameters and concentrations of ampicillin, IPTG and CuCl_2_ were maintained in the same proportion. Samples taken at regular intervals were analyzed for the presence of recombinant enzymes in all three fractions. In order to select the host producing maximum secretory recombinant enzymes, expression was also attempted in different *E*. *coli* strains viz. BL21(DE3), BL21(DE3) containing pTUM4 plasmid and BL21(DE3) arctic expression host. CPXpETDuet-1 construct was transformed into ultra-competent cells of these three hosts. Single colony of each transformant was processed for expression analysis in the same way except in case of BL21(DE3) arctic expression host, where pre-induction cultivation temperature was maintained as 30°C and post-induction temperature was maintained as 13°C. BL21(DE3)pTUM4 culture was supplemented with 34 μg/ml chloramphenicol along with ampicillin (100 μg/ml), whereas BL21(DE3) arctic expression host culture was provided with 20 μg/ml gentamycin, in addition to ampicillin (100 μg/ml). *E*. *coli* BL21(DE3) culture was supplemented with 100 μg/ml ampicillin only. After attaining OD_600nm_ up to 3.0, all three cultures were kept under static cultivation for 16h at respective temperatures. Thereafter, cultures were harvested and enzyme estimation was carried out in all fractions. Further for the sake of comparison, expression of individual protein was also carried out in BL21(DE3) cultivated TB media and production was compared between specific activities of all three recombinant enzymes produced during simultaneous and individual expression. During individual expression also, cultures were incubated under static condition for similar time periods. Comparison was recorded in terms of total extracellular and total intracellular protein fraction.

Samples withdrawn at regular intervals during expression and optimization were centrifuged at 10,000 x g for 10 min at 4°C and CSs were isolated. To extract PFs from the cell biomass (1.25 ml culture), pellets were resuspended in 1 ml (10% w/v) sucrose solution prepared in 50 mM Tris HCl (pH 8.0) containing 5 mM EDTA. The cell suspensions were incubated at 25°C and 200 rpm for 15 min followed by centrifugation at 10,000 x g for 10 min at 4°C. Pellets were resuspended in 250 μl 5 mM MgSO_4_ and kept in ice for 10 min to release PF, while, cytoplasmic protein fractions were extracted by sonicating spheroplasts obtained from the previous step in 50 mM Tris HCl buffer (pH 8.0) programmed as 10 s pulse ON and 20 s pulse OFF for a total pulse time of 3 min in ice bath. Culture lysates were centrifuged at 12,000 x g, 4°C for 20 min and CF were isolated. CS, PF and CF were used for enzyme estimation using standard methods given below.

### Enzyme activity assays

Activity of CotA was determined as described earlier [10] using 1 mM 2,2′-azinobis-(3-ethylbenzothiazoline-6-sulfonic acid) as substrate prepared in 100 mM citrate phosphate buffer (mixture of 0.1 M citric acid and 0.2 M K_2_HPO_4_) of pH 4.8. Oxidation of ABTS (Ɛ = 36,000 M^-1^cm^-1^) was measured at 25°C in a reaction volume composed of 900 μl of substrate and 100 μl of enzyme (appropriately diluted) by time scan of 1 min at 420 nm. One unit of enzyme activity was equivalent to the amount of enzyme required to oxidize 1 μmol of substrate per minute. Pectate lyase activity was determined according to the method described earlier [11]. Polygalacturonic acid (0.1% w/v) prepared in 50 mM glycine NaOH buffer (pH 9.0) was used as substrate. Reaction was set up by incubating 900 μl of substrate and 100 μl of pectate lyase at 60°C for 10 min. Substrate degradation (Ɛ = 4600 M^-1^cm^-1^) was analyzed by taking absorbance at 235 nm. One unit of enzyme activity was defined as the amount of enzyme required to produce 1 μmol of unsaturated oligogalacturonide per minute at 60°C [11]. Endo-xylanase activity was determined by following the method described earlier [3]. Substrate was prepared at 1.0% (w/v) concentration in 100 mM citrate phosphate buffer (pH 5.0). Reaction mixture composed of 490 μl substrate solution and 10 μl enzyme dilution was incubated at 50°C for 10 min. Release of the reducing sugars was measured by dinitrosalicyclic acid method [12]. One unit of xylanase activity was defined as the amount of enzyme that released 1 μmol of reducing sugar equivalent to xylose per minute. Total protein concentration was determined by Bradford’s method using bovine serum albumin (BSA) as standard.

### *In silico* analysis of proteins

Amino acid sequences of all the proteins were retrieved from the protein database available on NCBI website and compared with the translated sequence of cloned genes using pair wise alignment (http://www.ebi.ac.uk/Tools/msa/clustalw2/). The sub-cellular location of cloned gene product was assessed i*n silico* using SignalP 4.0 [13] and PSORTb 3.1 [14]. In addition, homology based detection of CotA sequence was carried out using NCBI-BLASTp tool against SwissProt database. Results of *in silico* analysis were compared with the subcellular localization of expressed recombinant enzymes.

### Scale up of enzyme production using *E*. *coli* BL21(DE3)

#### At flask level

Recombinant enzyme production was scaled up from 20 ml culture media in 100 ml Erlenmeyer flask to 400 ml cultures raised in 2 L flasks. At each level, TB medium was inoculated with 2% of seed culture and flasks were incubated at 37°C and 200 rpm before induction and at 25°C, 200 rpm post induction for 8 h followed by static cultivation for 16 h at same temperature. Cultures were induced with 0.6 mM IPTG and 0.25 mM CuCl_2_.

#### At bioreactor level

After optimizing physiological and nutritional parameters at flask level, enzymatic cocktail was produced in 3 L working volume bioreactor (Electrolab Biotech., UK). *E*. *coli* BL21(DE3) transformant was inoculated in 15 ml LB ampicillin (100 μg/ml) medium in 100 ml Erlenmeyer flask and cultivated overnight at 37°C, 200 rpm. To prepare inoculum for fermenter, fresh 100 ml TB was inoculated by 5 ml of overnight grown culture and allowed to grow upto OD_600nm_ ~3. This inoculum was used to inoculate 900 ml modified TB medium (20 g/L glycerol) in 3 L bioreactor and incubated at 37°C and DO was maintained at 40% through cascading in agitation range from 250–700 rpm, whereas pH 7.2 was maintained using 4 N NaOH and 4 N HCl. Optimum induction OD was determined for maximum biomass production by inducing the culture at different optical density in different batches. Culture was induced with 0.6 mM IPTG and supplemented with 0.25 mM CuCl_2_. Post-induction bioreactor temperature was maintained at 25°C in order to avoid improper folding of the proteins. Medium containing (% w/v) 20% D-glucose, 10% yeast extract and 10% peptone were used as feed which was added at a flow rate corresponding to change in concentration of C-source (glycerol and D-glucose) which was monitored throughout run by metabolic analyzer (YSI Biochemistry analyzer, USA). Growth rate and production level of recombinant enzymes was measured by taking out samples at regular interval of 1 h.

### Saccharification efficiency of the recombinant cocktail

Recombinant enzyme cocktail was used for the hydrolysis of orange peel a pectin containing lignocellulosic substrate. The substrate was dried in hot air oven at 50°C for 36 h, grinded and sieved to mesh size 40. Compositional analysis was done by following TAPPI method (1992) [15]. Saccharification was carried out in 50 mM citrate phosphate buffer pH 6.5 at 60°C by maintaining substrate to buffer ratio of 1:10 (w/v). Treatment of 1 g substrate was carried out by 2 different enzyme combinations viz. (i) CPX cocktail having 51 IU/g of CotA, 1050 IU/g of xylanase, and 1731 IU/ml of pectate lyase; and (ii) xylanase alone having 1050 IU/g activity. Saccharification was carried out for 30 h while samples were withdrawn in every 6 h and assayed for sugar release, galacturonic acid release and phenolics. Sugar release was estimated by using DNSA method [12], galacturonic acid release was estimated by spectrophotometric detection at 235 nm [11] and phenolics release were detected by Singleton method [16].

## Results and Discussion

### Synthesis of CPXpDuet-1 plasmid

**Fig 2:**
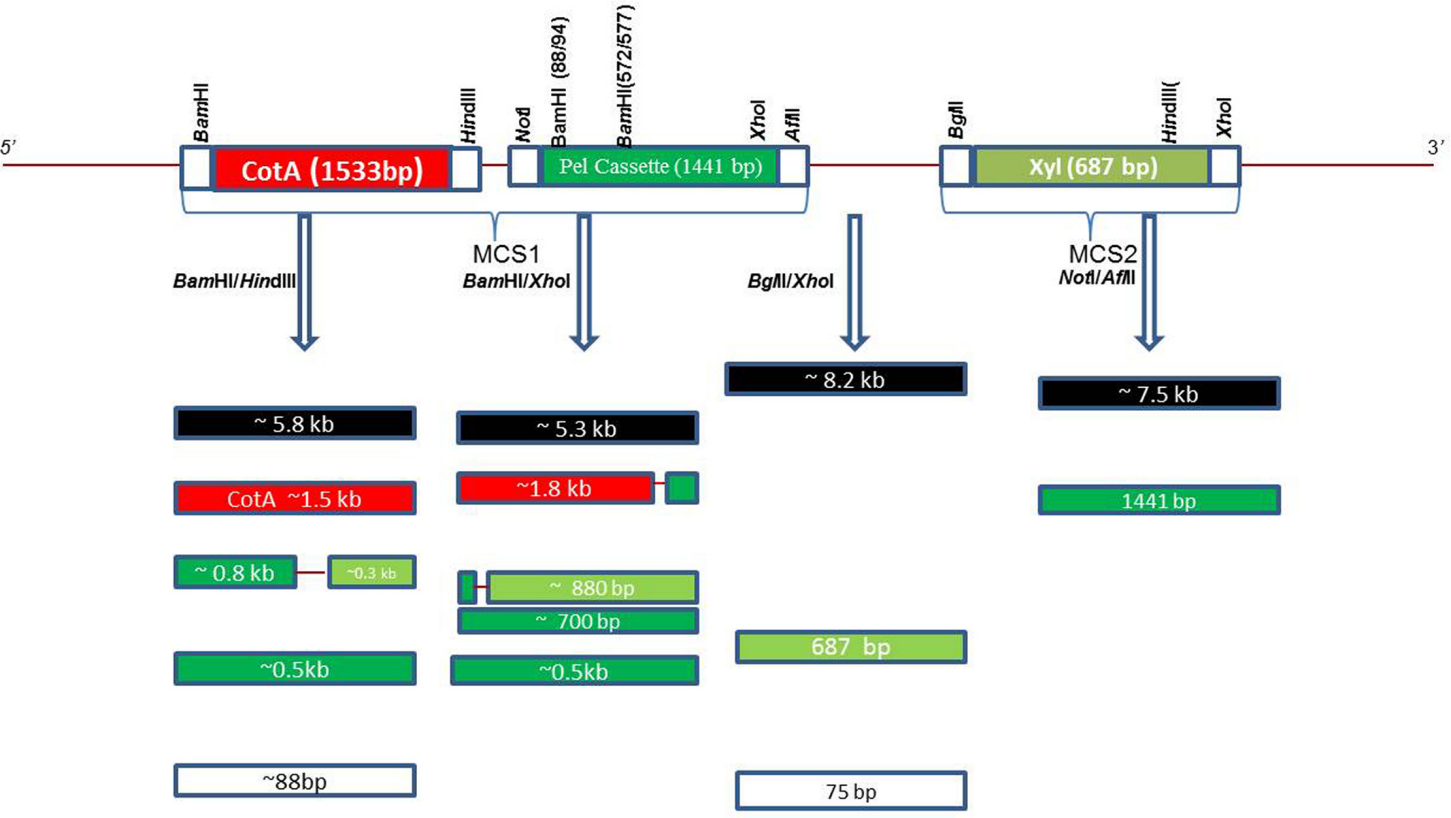
Predicted restriction digestion pattern of CPXpETDuet-1 plasmid construct when treated with different restriction enzyme combinations.

The expression of multiple genes in a single host is one of the most fascinating approaches for the cost effective production of two or more enzymes simultaneously. The present work was carried out to develop a system capable of producing laccase, xylanase and pectinase in a single host for their cost effective production. pETDuet-1, a medium copy number vector, which contains pBR322 derived ColE1 replicon and has multiple cloning sites for two genes in its native form, was used for cloning purpose. Accuracy of ligation of all the genes was confirmed by restriction digestion followed by nucleotide sequencing. Restriction digestion pattern was obtained as per the presence of restriction sites when the plasmid construct was digested with different combinations of restriction enzymes (Fig 2). Nucleotide sequencing and restriction map analysis of each gene revealed the presence of two *Bam*HI recognition sites in *pel* ORF and one *Hin*dIII recognition site in *xyl* ORF which resulted in visualization of five bands after digestion with *Bam*HI/*Hin*dIII restriction enzymes. *Pel* gene cassette was also detected to have one *Xho*I recognition site, therefore, resulted in visualization of five bands after digestion by *Bam*HI/*Xho*I restriction enzymes (data not shown).

### Optimization of expression media and host

**Fig 3:**
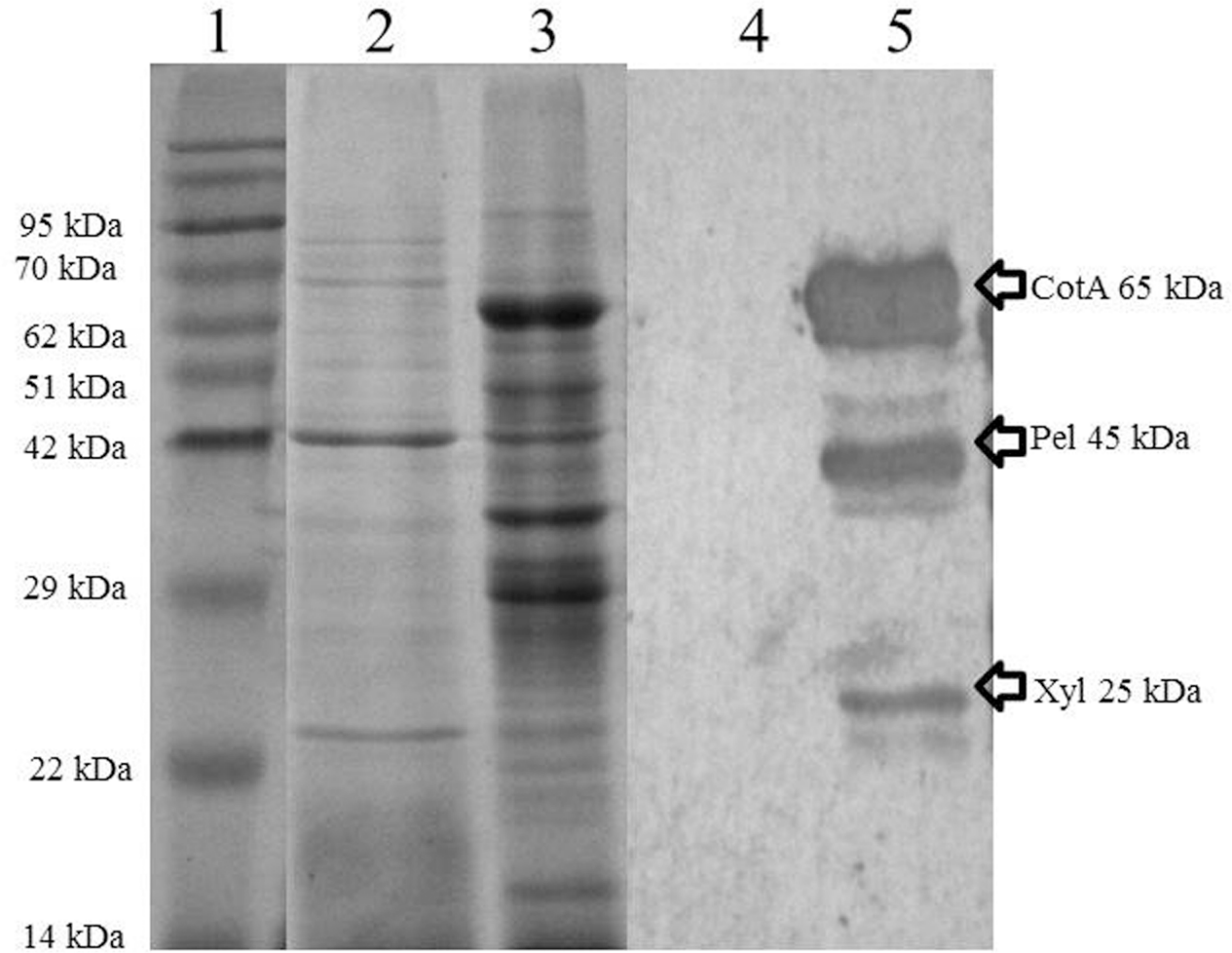
Western blotting analysis of the expression of recombinant cocktail in *E*. *coli* BL21(DE3). Lane 1: Protein MW marker; Lane 2: Supernatant of induced culture; Lane 3: Cell biomass of induced culture; Lane 4–5: Western blot of CS and cell biomass showing no signal in the supernatant which might be due to removal of N-terminal signal sequence along with His tag during secretion.

In shake flask studies, simultaneous expression of all three recombinant proteins was observed by SDS-PAGE which revealed the presence of distinct proteins bands of size 65 kDa, 45 kDa and 23 kDa. The expression was further confirmed by western blot (Fig 3). Continuous increase in biomass was observed till 8 h post-induction. Under static condition no further increase in biomass was observed. The culture was kept static to maintain microaerophilic conditions for efficient copper incorporation in the CotA tertiary structure. This enzyme reportedly requires minimum oxygen concentration for being fully charged with Cu^2+^, which is required as cofactor for enzyme activity [17]. Interestingly, a significant amount of all three enzymes was also observed in secretory fraction; therefore, activities of all three recombinant enzymes in sub-cellular fractions were analyzed. Static cultivation was certainly responsible for decreasing total protein yield, however, a significant increase in secretion of all three enzymes was also observed. Similar effect of static cultivation on the secretion of recombinant protein has also been reported earlier [18]. Increased leakage of the recombinant proteins during oxygen limiting conditions has been reported due to change in the porosity of the outer membrane as a result of change in outer membrane protein and lipid composition [19].

**Fig 4:**
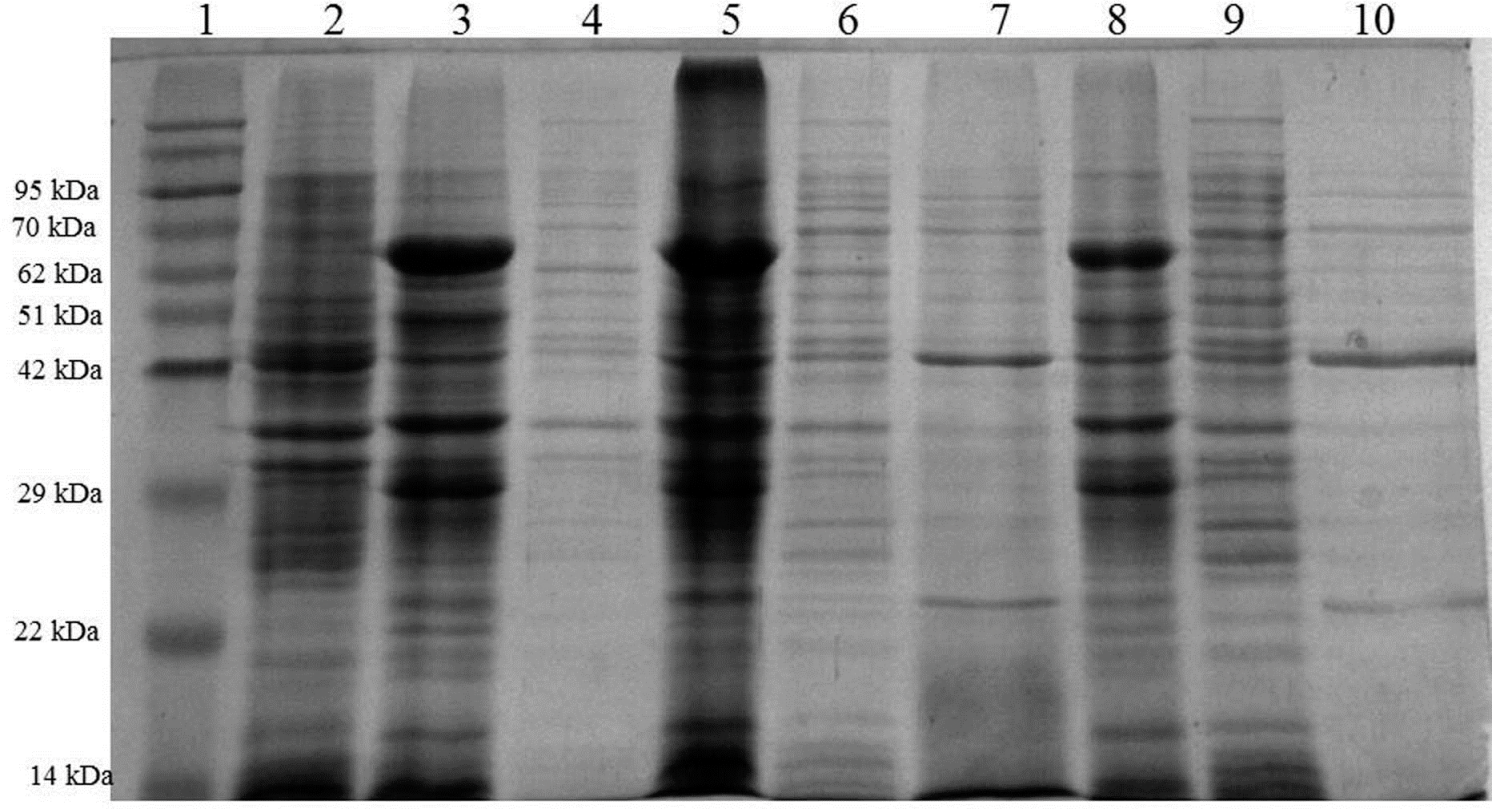
Comparison of CPX expression when cultivated in LB, wheat bran and terrific broth. Significant amount of pectate lyase and endoxylanase was observed in CS. Lane 1: Protein MW marker; Lane 2: LB uninduced control culture; Lane 3–4: LB induced culture cell biomass and CS; Lane 5–7: LB+wheat bran induced culture CF pellet, CF sup and CS; Lane 8–10: terrific broth induced culture CF pellet, CF sup and CS.

All three enzymes showed variability in their activities based on post-translation sub-cellular localization. CotA showed maximum activity as CF, Xyl was observed as maximum in the CS and Pel was found mainly in CS and PF. In comparison to LB medium (S2 Fig), cultivation of *E*. *coli* BL21(DE3) in LB supplemented with 1% (w/v) wheat bran (S3 Fig) and TB (S4 Fig) resulted in increased activities of all recombinant enzymes in all the fractions. Cultivation in LB wheat bran medium produced 238 IU/ml, 5 IU/ml and 250 IU/ml activities of Pel, CotA and Xyl, respectively, after 8 h of induction, and 596 IU/ml, 13 IU/ml and 265 IU/ml, respectively, after 16 h of static incubation in the extracellular fraction (S3 Fig). On the other hand, cultivation in TB supplemented medium resulted in production of 580 IU/ml, 9 IU/ml and 222 IU/ml activities of Pel, CotA and Xyl, respectively, after 8 h of induction, and 681 IU/ml, 16 IU/ml and 371 IU/ml, respectively, after 16 h of static incubation in the extracellular fraction (S4 Fig). Both wheat bran and terrific broth are rich carbon source for the growing culture which subsequently helps in log phase prolonging. Presence of wheat bran in the growth medium significantly escalated the activity of pectate lyase in the CS to a significant degree in comparison to endoxylanase and CotA. Terrific broth contains tryptone, yeast extract and phosphate buffer, which is reported to enhance the cell density and consequently the total protein yield [20]. Conclusively, LB wheat bran medium resulted in maximum enzyme production in CF whereas, terrific broth was observed to stimulate maximum production of all three recombinant enzymes as secretory fraction; therefore, this medium was used for further production. The expression levels were also compared on the SDS gel and results were in accordance with the enzyme activities obtained in all the fractions (Fig 4).

Formation of inclusion bodies (IBs) is a common phenomenon when forced expression of heterologous protein is carried out in *E*. *coli*. Cultivation of the host cells at suboptimal temperature and co-expression of refoldases along with recombinant protein, are two major remedies taken to minimize the possibilities of IBs formation [21]. To take advantages of these technologies, *E*. *coli* BL21(DE3) arctic and BL21(DE3)pTUM4 host were tested along with BL21(DE3). From 15 ml culture of *E*. *coli* strain BL21(DE3), BL21(DE3)pTUM4 and BL21(DE3)Arctic, ~4.16 mg, ~3.4 mg and ~4.5 mg total cellular protein was obtained, respectively. Recombinants expressed in arctic host resulted in maximum amount of cytoplasmic protein, however, total secretory fraction reduced significantly (Table 2). Expression of CotA, Pel and Xyl in BL21(DE3) resulted in production of maximum secretory enzymes followed by BL21(DE3) Arctic and BL21(DE3) pTUM4. Extracellular activity of CotA, Pel and Xyl was estimated 17 IU/ml, 685 IU/ml, 223 IU/ml in BL21(DE3), 3.7 IU/ml, 275 IU/ml, 102 IU/ml in BL21(DE3)arctic and 0.8 IU/ml, 651 IU/ml, 179 IU/ml inBL21(DE3)pTUM4, respectively. However, the maximum activity of all the enzymes in cytoplasmic fraction was obtained in BL21(DE3) Arctic. While in PF, the enzyme activity of CotA and Pel was found maximum in BL21(DE3), enzyme activity of Xyl was maximum in BL21(DE3) Arctic. *E*. *coli* BL21(DE3) arctic expression host is engineered to express chaperonins Cpn10 and Cpn60 taken from *Oleispira antarctica*. These chaperonins are 74% and 54% amino acid identical respectively to the *E*. *coli* GroEL and GroES and show high protein refolding activities at lower temperatures (4 to12°C) [21]. *E*. *coli* BL21(DE3)pTUM4 cells harbor helper plasmid pTUM4, which assist in hyper expression of periplasmic chaperones: thiol-disulfide oxidoreductases DsbA and DsbC, peptidyl-prolyl cis/trans-isomerases, FkpA and SurA, which subsequently help in proper folding of heterologous proteins [22]. Variability in the localization of all three recombinants was recorded during expression in different hosts; however, no specific justification for this deferential behavior could be derived from the observations.

**Table 2:** Activity of CotA (a), Xyl (b) and Pel (c) in CS, PF and CF when expressed in *E*. *coli* BL21(DE3), *E*. *coli* BL21(DE3) pTUM4 and *E*. *coli* BL21(DE3) Arctic.

Host	CotA activity (IU/ml)			Pel activity (IU/ml)			Xyl activity (IU/ml)		
	CS	PF	CF	CS	PF	CF	CS	PF	CF
**BL21(DE3)**	17.06±0.85	5.64±0.45	133.43±5.8	685.25±8.2	445.2±11.6	100.8±2.1	223.4±5.6	51.5±1.23	42.4±2.24
**BL21(DE3)pTUM4**	0.78±0.031	2.56±0.16	168.9±3.9	651.87±9.1	199.5±3.1	10.2±0.21	179.5±5.2	54.5±1.5	85.6±3.7
**BL21(DE3)Arctic**	3.71±0.12	4.0±0.26	181.3±5.07	275.53±4.68	154.1±4.5	962.7±9.8	102.3±1.6	95.8±3.3	168.9±5.6

Activity of various fractions was calculated by first extracting periplasm from biomass of 1.25 ml culture resuspended in 1 ml extraction buffer. Spheroplast obtained after periplasmic fluid extraction was resuspended in 1 ml lysis buffer and processed for sonication.

### *In silico* analysis of the proteins

SignalP and PSORTb detection of amino acid sequences revealed the presence of secretory signal sequences in Pel and Xyl (S5 Fig). It is speculated that the recognition of the signal sequences present in Pel and Xyl by *E*. *coli* could be the reason for their extracellular secretion. Pel has 21 N-terminal residues coding for secretory signal sequence with a total D-score of 0.909 as per SignalP detection. A score beyond 0.570 is considered as significant for secretory nature of a peptide. PSORTb detection also predicted the presence of non-cytoplasmic signal sequence with a score of 10. Similarly, for Xyl, 27 N-terminal residues were predicted as signal sequence by SignalP with a D-score of 0.858. PSORTb also noticed the presence of a non-cytoplasmic signal sequence in the protein, however, final prediction was observed as unknown for the protein. In contrast to both these proteins, CotA was not observed to harbor any signal sequence, which make it difficult to draw any firm conclusion. Specific activity of Pel and Xyl was recorded as higher in total during individual expression than in the cocktail, however, in CS activity was observed as slightly less. CotA also exhibited comparatively higher activity in total, however, CS was observed as devoid of laccase activity during individual gene expression (Table 3). A definite conclusion could not be drawn from the results; however, higher specific activities of recombinant proteins during individual expression may be due to low burden experienced by the cell in comparison to multiple gene expression [23]. Mutual interaction between recombinant proteins has been reported as one of the factor responsible for cellular behavior. Expression of individual protein shows less dependence on other factors which consequently enhance the solubility of the protein [23]. Therefore, it may be speculated that simultaneous expression of three foreign proteins enforces the cell to secrete extra proteins. Interestingly, the sub-cellular localization revealed the presence of all the enzymes in cytoplasm, periplasmic space and culture filtrate. This led us to purpose that all these proteins are possibly being transferred out of the cell in a two-step process, i.e. first to the periplasmic space and then excreted to the medium. Possibly, the complete process is accomplished by type II secretion mechanism [24] as type I secretion system in *E*. *coli* has been reported as inefficient in secretion of proteins composed of more than 200 amino acid residues [25].

**Table 3:** Comparison of the Activities of Pel, CotA and Xyl between cocktail and individual gene expression.

Fraction	Specific Activity in cocktail (IU/mg)			Specific Activity during individual expression (IU/mg)		
	CotA	Pel	Xyl	CotA	Pel	Xyl
**Secretory**	33±1.6	755±8.6	790±7.2	nil	719.1±12.02	748.2±9.3
**Intracellular**	34.8±2.1	48±1.12	18.76±0.9	109.8±2.7	154.6±7.4	137.25±4.8

Specific activity was calculated as per enzyme activity and protein concentration recorded in 1 ml sample. Comparison of the activities was measured in reference to total extracellular and intracellular protein estimation.

### Scale up of enzyme production using *E*.*coli* BL21(DE3)

Recombinant cultivation in bioreactor provides better control of the process parameters in comparison to shake flask cultivation. Maximum titer of recombinant enzymes was recorded when grown in TB; therefore, further scale up was carried out in the same medium. The production of enzymes was efficiently scaled up to 20 ml, 50 ml, 100 ml, 200 ml and 400 ml culture medium cultivation in 100 ml, 250 ml, 500 ml, 1 L and 2 L Erlenmeyer flasks, respectively (Table 4). When 400 ml medium was cultivated in 2 L Erlenmeyer flask, enzyme activities in CS, PF and CF were recorded as 615 IU/ml, 416 IU/ml and 62 IU/ml, respectively, of Pel, 14 IU/ml, 4 IU/ml and 137 IU/ml, of CotA, and 330 IU/ml, 58 IU/ml and 15 IU/ml of Xyl after 12 h static incubation which was comparable to enzyme production at 100 ml flask level.

**Table 4:** Activity of recombinant enzymes from various sub-cellular fractions obtained during scale-up.

Volume (ml)	CotA activity (IU/ml)			Xyl activity (IU/ml)			Pel activity (IU/ml)		
	CS	PF	CF	CS	PF	CF	CS	PF	CF
20	15.9±1.43	4.8±0.23	148.5±2.3	370.7±10.3	77.2±2.8	16.8±1.3	724.4±23.4	513.0±12.3	110.4±1.2
50	15.3±.123	4.4±0.53	143.2±3.2	359.8±21.4	70.0±1.2	13.9±2.1	699.1±21.4	484.9±30.1	98.2±4.5
100	15.1±0.95	4.3±1.2	141.8±4.1	351.4±8.4	63.7±3.6	14.9±0.43	656.2±12.8	476.2±12.9	86.7±6.2
200	14.8±1.21	4.2±0.96	140.3±5.3	343.9±9.2	59.4±2.4	12.6±0.56	648.6±16.8	441.0±11.3	83.5±5.3
400	14.5±0.65	4.0±0.32	137.1±8.4	333.8±5.4	57.9±1.2	15.2±0.87	615.6±12.4	416.4±20.3	62.2±7.1

Activity of various fractions was calculated by first extracting periplasm from biomass of 5 ml culture resuspended in 1 ml extraction buffer. Spheroplast obtained after periplasmic fluid extraction was resuspended in 1 ml lysis buffer and processed for sonication.

**Fig 5:**
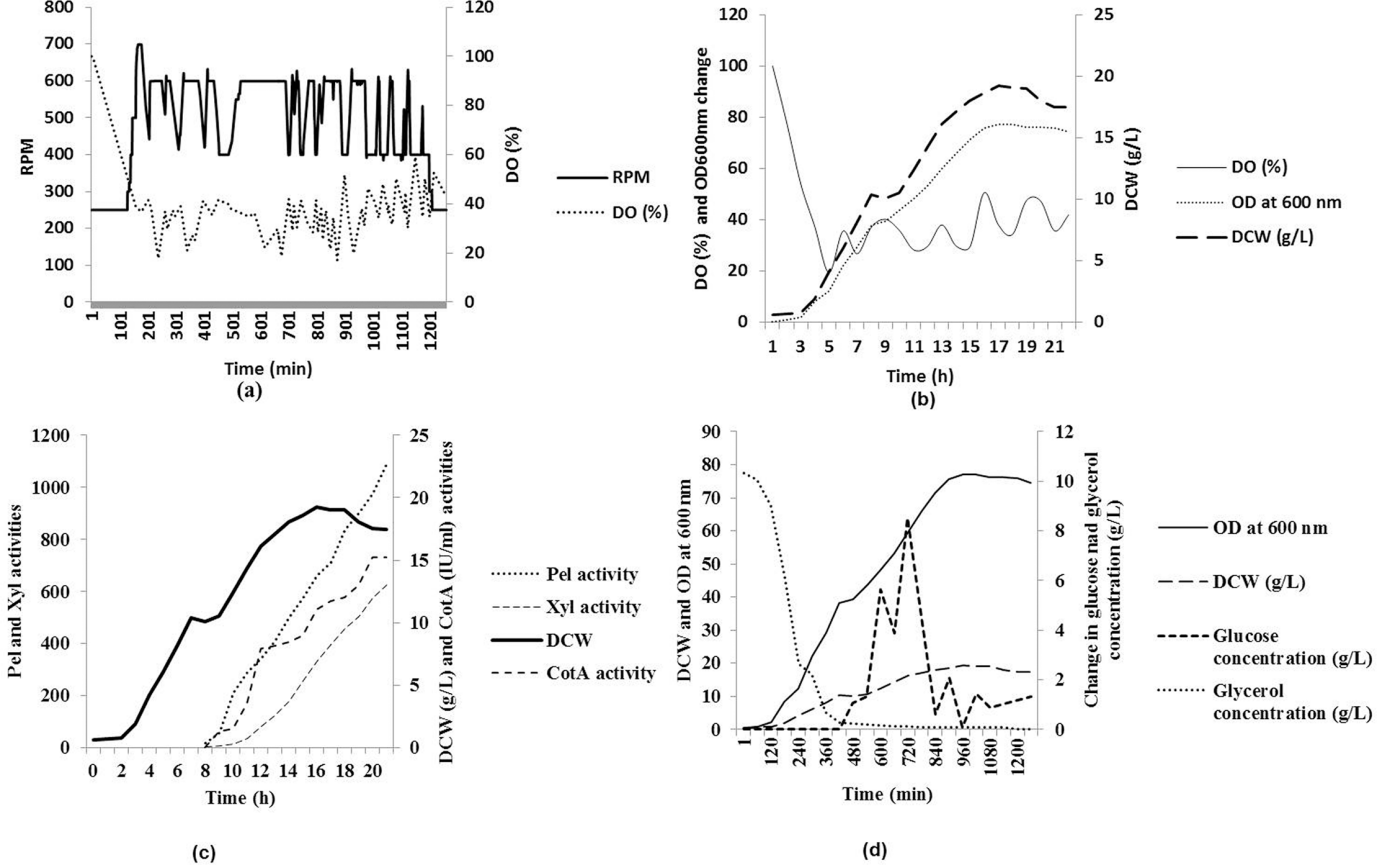
Bioreactor level optimization of the recombinant enzyme cocktail production. (a) Change in DO concentration with respect to change in RPM; (b) Increase in dry cell weight (DCW) with respect to change in OD_600nm_, and DO concentration; (c) Increase in the activities of CotA, Pel and Xyl with respect to DCW; and (d) Change in concentration of glucose and glycerol with respect to DCW and OD_600nm_.

At bioreactor level, induction at OD_600nm_ ~29 resulted in maximum productivity in terms of specific productivity. Optimization of the induction time affects the final yield of biomass as well as recombinant proteins. Increase and decrease in the biomass yield with respect to time of induction has been reported in literature [26]. Growth rate of the culture decreased from 0.23 h^-1^ to 0.13 h^-1^ after induction. Growth of the culture became static after 14 h of cultivation and slight decrease in OD and DCW was recorded after 16 h, however, activity of Pel and Xyl were observed to increase upto 21 h of cultivation. Culture was harvested at OD_600nm_ ~74 from which ~19 g DCW was obtained (Fig 5). In the secretory fraction, maximum activity of Pel and Xyl were recorded as 1090 and 627 IU/ml, respectively, after 7 h of induction, whereas, maximum activity of CotA was estimated as 9 IU/ml (Fig 5), which was significantly lesser than observed during shake flask cultivation. It may be attributed to non-inclusion of static cultivation during fermentation process, which is required for proper incorporation of Cu^2+^ ions in the enzyme active site [17]. However exact comparison of this expression study to the various studies which have need done using single gene expression could not been done directly, but the expression levels of all the enzymes were comparable to the previous reports [6, 10, 17, 27, 28].

### Saccharification efficiency of the recombinant enzyme cocktail

**Fig 6:**
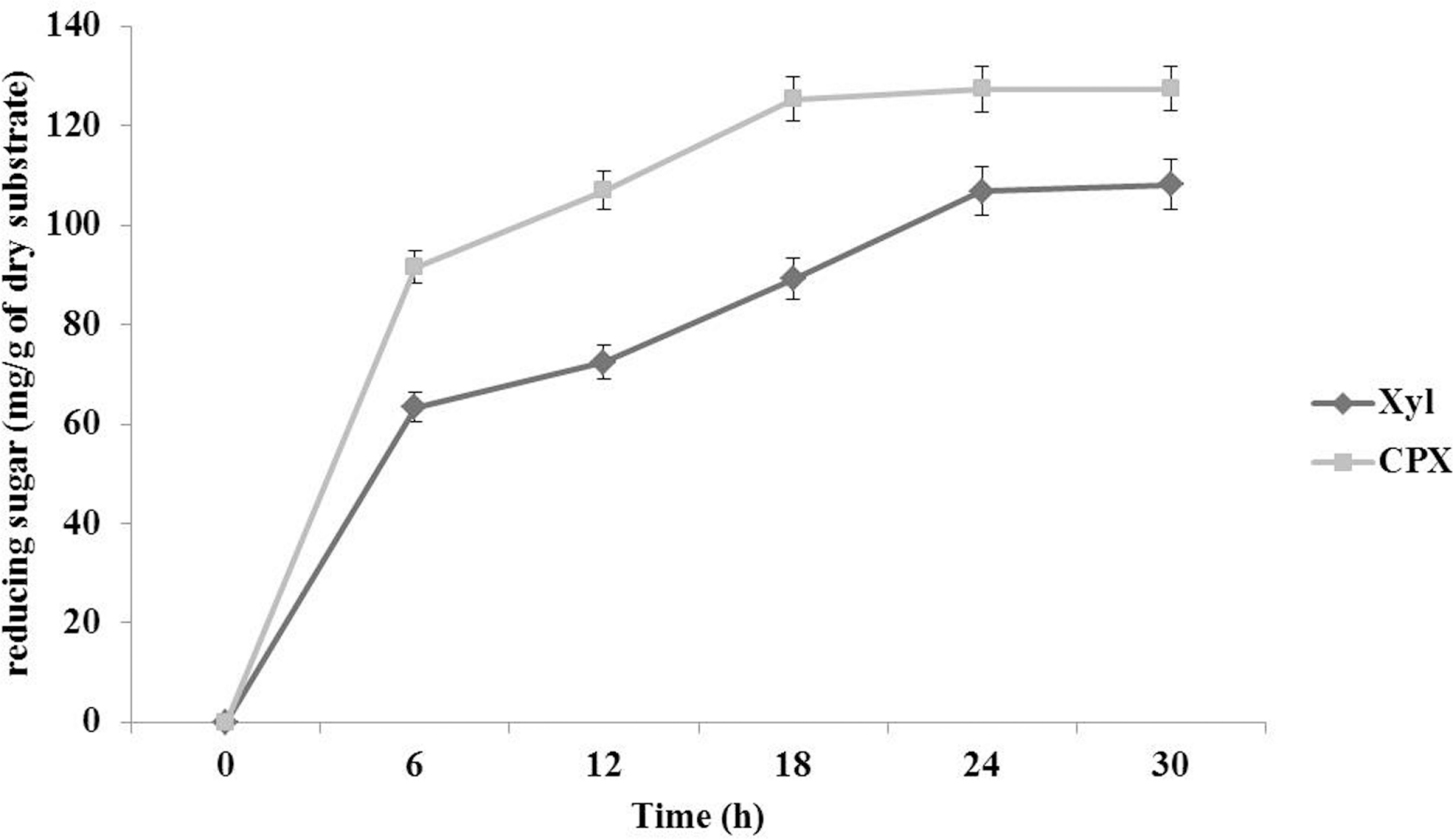
Comparison of the hydrolyzing efficiency of CPX enzyme cocktail and Xyl alone by sugar release estimation at uniform time intervals.

Recombinant enzyme cocktail was observed to be efficient in saccharification of orange peel taken as lignocellulosic substrate. Being a rich source of cellulose, hemicellulose, protein and pectin in dried orange peel with a low nutritional content supports its suitability as a potential substrate for biofuel production [29]. Continuous increase in sugar release was observed upto 24 h. CPX cocktail resulted in fast release of the sugars in comparison to Xyl alone. A total of 127.38 mg/g dry substrate sugar was release by CPX in comparison to 106.85 mg/g dry substrate after 24 h of incubation. Beyond 24 h no further increase in sugar release was observed (Fig 6). In addition to reducing sugars, release of some phenolic compounds (5.4 mg/g) and D-galacturonic acid (13.0 mg/g of dry substrate) was also observed which was specifically catalyzed by CotA and Pel, respectively. This increase in sugar may be attributed to the synergistic action of all the three enzymes, thus lowering the need of chemical pretreatment for the hydrolysis. This may help in reducing the requirements of chemicals which are harmful to the environment and also releases inhibitory compounds for the downstream applications [30]. The amount of reducing sugar released in the present study by the use of enzyme cocktail was comparable to the earlier reports [31, 32].

## Conclusion

This study has attempted to exhibit the potential for developing technology for cost-effective production of desired cocktail of lignocellulolytic enzymes in a single host. This enzyme cocktail would be of immense value in pretreating the lignocellulosic residues for production of biofuel. While there is scope for further improvement in this strategy for its optimum utilization, this study has laid the foundation for the production of other industrially important enzymes in a similar manner. Moreover, secretion of the recombinant enzymes using expression vector developed in this study significantly increases the economic viability of this work.

## Supporting Information

S1 Fig Construction strategy of CPXpETDuet-1 plasmid. (TIF)

S2 Fig Activity of CotA, Xyl and Pel in CS (a), PF (b) and CS (c) when cultivated in Luria Bertani broth. (TIF)

S3 Fig Activity of CotA, Xyl and Pel in CS (a), PF (b) and CF (c) when cultivated in Luria Bertani broth + 1% wheat bran. (TIF)

S4 Fig Activity of CotA, Xyl and Pel in CS (a), PF (b) and CF (c) when cultivated in terrific broth. (TIF)

S5 Fig Detection of the presence of signal sequence in amino acid sequences of (a) Pel (b) Xyl and (c) CotA. (TIF)
