# Supplementary figures and images for: Multiple Genes in a Single Host: Cost-Effective Production of Bacterial Laccase (cotA), Pectate Lyase (pel), and Endoxylanase (xyl) by Simultaneous Expression and Cloning in Single Vector in E. coli
Source: PLoS One. 2015 Dec 7;10(12):e0144379. doi: 10.1371/journal.pone.0144379 (PMC4671577; doi:10.1371/journal.pone.0144379)

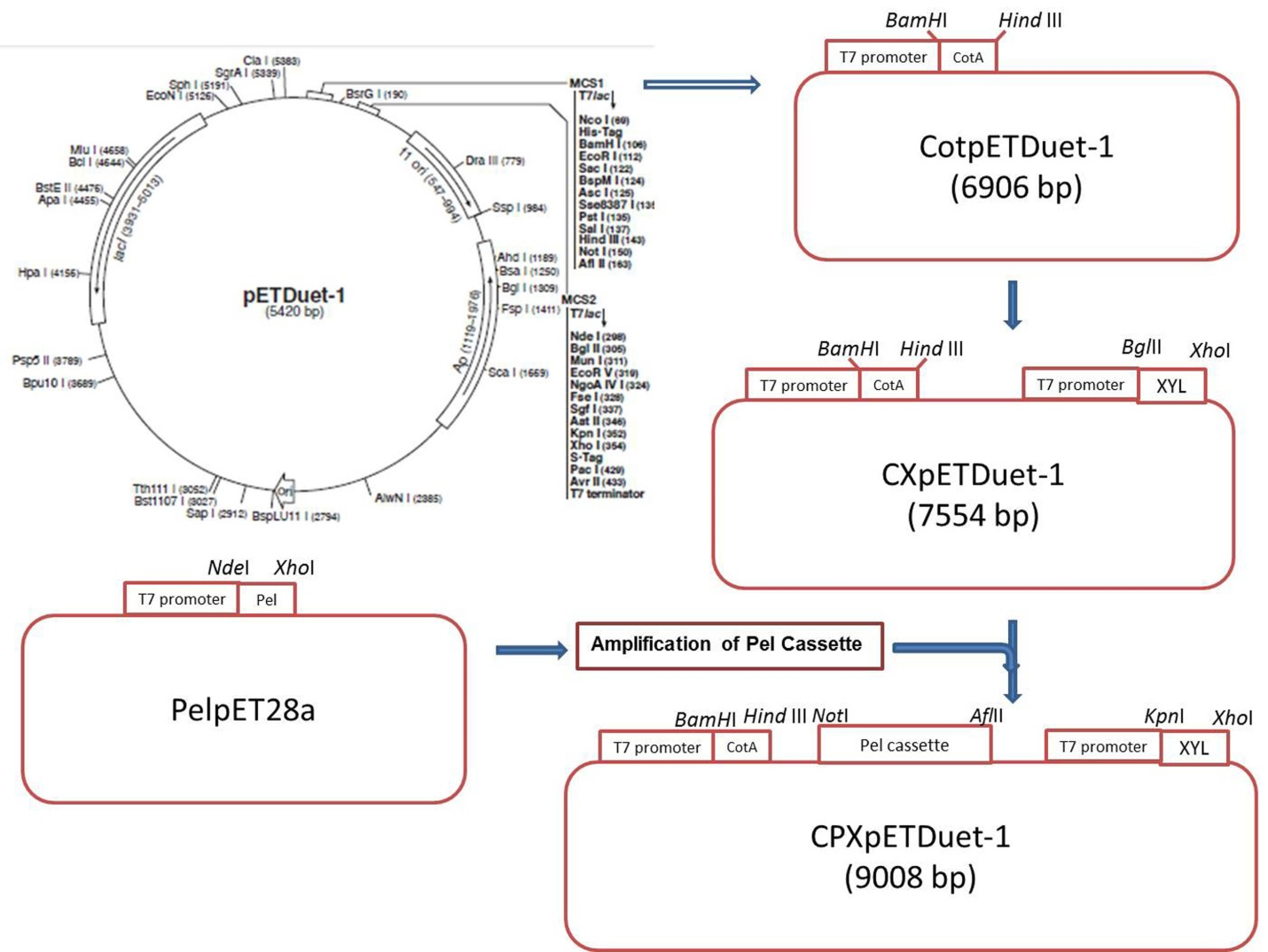

Supplement: S1 Fig — (TIF) [file pone.0144379.s001.tif]

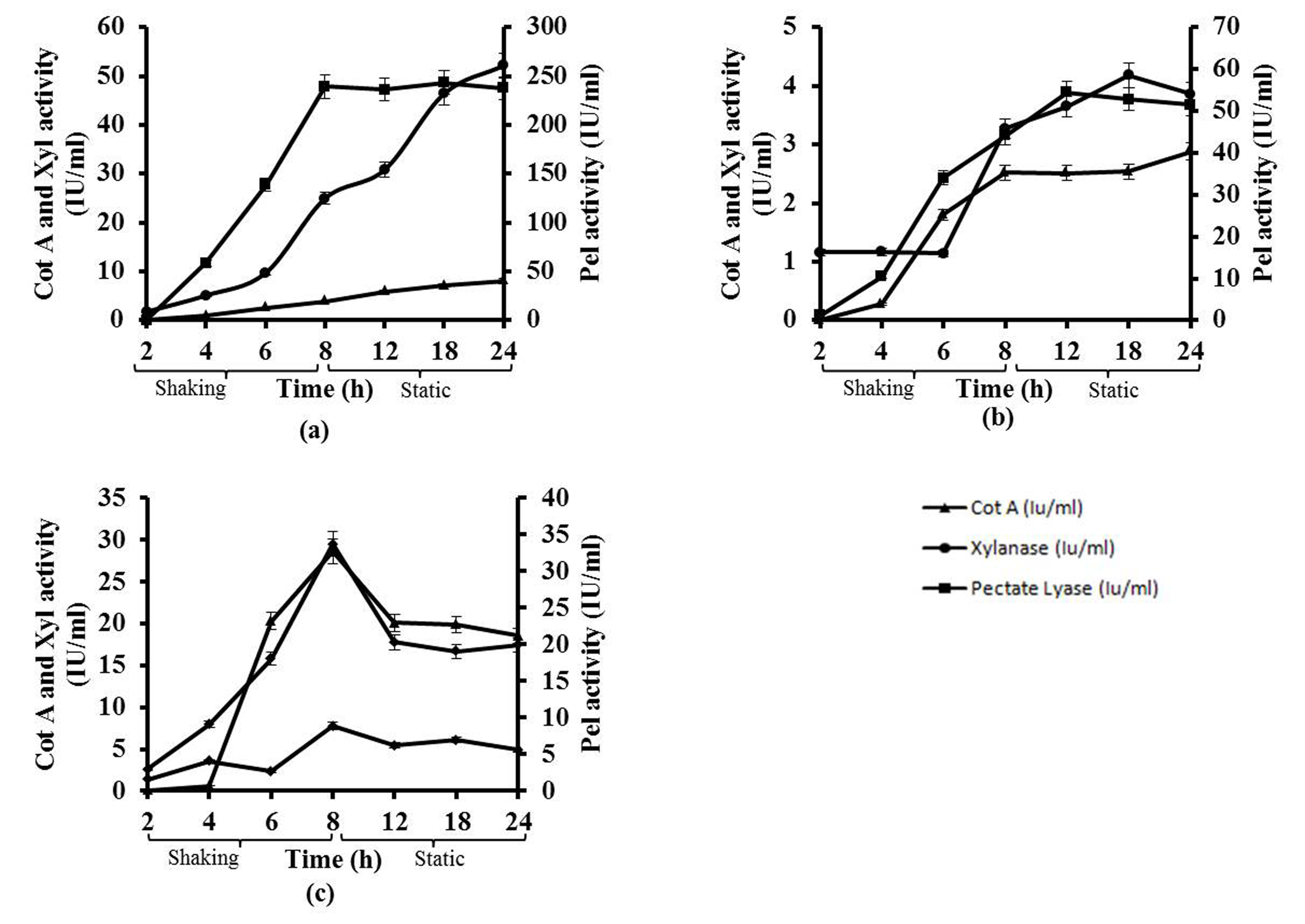

Supplement: S2 Fig — (TIF) [file pone.0144379.s002.tif]

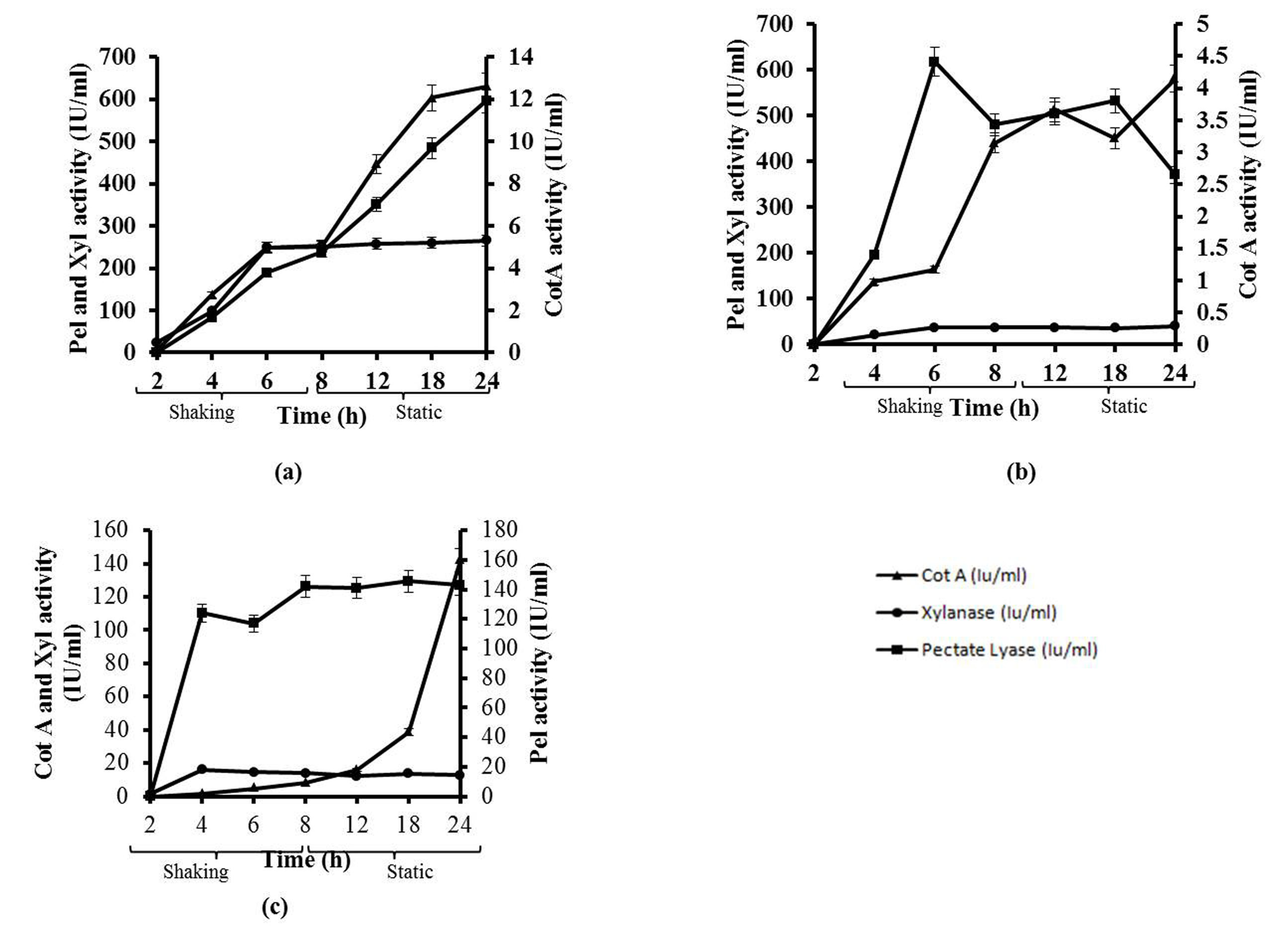

Supplement: S3 Fig — (TIF) [file pone.0144379.s003.tif]

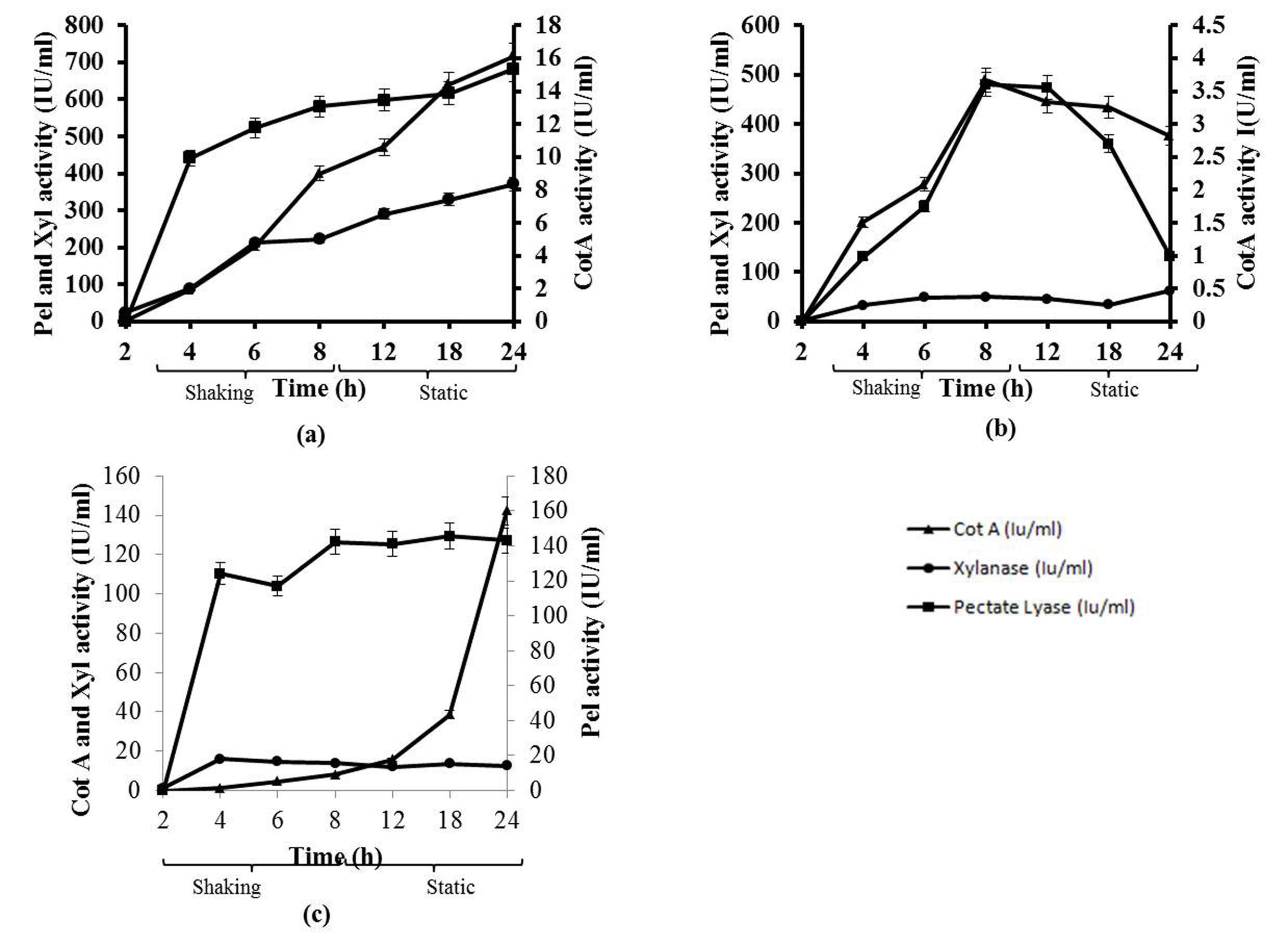

Supplement: S4 Fig — (TIF) [file pone.0144379.s004.tif]

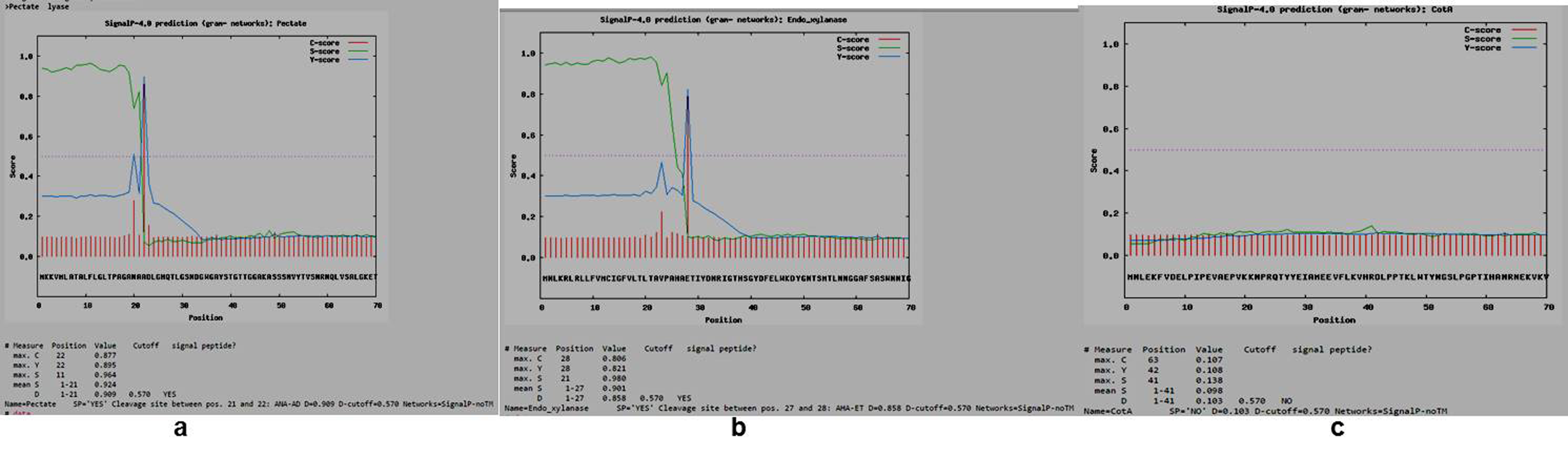

Supplement: S5 Fig — (TIF) [file pone.0144379.s005.tif]
